# First translational ‘Think Tank’ on cerebrovascular disease, cognitive impairment and dementia

**DOI:** 10.1186/s12967-016-0806-z

**Published:** 2016-02-13

**Authors:** Frank C. Barone, Deborah Gustafson, Howard A. Crystal, Herman Moreno, Mateusz G. Adamski, Ken Arai, Alison E. Baird, Clotilde Balucani, Adam M. Brickman, David Cechetto, Philip Gorelick, Geert Jan Biessels, Amanda Kiliaan, Lenore Launer, Julie Schneider, Farzaneh A. Sorond, Rachel Whitmer, Clinton Wright, Zheng Gang Zhang

**Affiliations:** Neurology, SUNY Downstate Medical Center, Brooklyn, NY USA; Physiology and Pharmacology, SUNY Downstate Medical Center, Brooklyn, NY USA; Section Neuroepidemiology, SUNY Downstate Medical Center, Brooklyn, NY USA; Pathology, SUNY Downstate Medical Center, Brooklyn, NY USA; Jagiellonian Centre for Experimental Therapeutics, Jagiellonian University, Krakow, Poland; Neuroprotection Research Laboratory, Departments of Radiology and Neurology, Massachusetts General Hospital, Harvard Medical School, CharlesTown, Boston, MA USA; Taub Institute for Alzheimer’s Disease and the Aging Brain, Department of Neurology, Columbia University, New York, NY USA; Department of Anatomy and Cell Biology, Schulich School of Medicine and Dentistry, Western University, London, ON Canada; Translational Science and Molecular Medicine, Michigan State University College of Human Medicine, Mercy Health Hauenstein Neurosciences, Grand Rapids, MI USA; Department of Neurology, Brain Center Rudolf Magnus, University Medical Center Utrecht, Utrecht, The Netherlands; Department of Anatomy, Preclinical Imaging Center, Donders Institute for Brain, Cognition and Behaviour, Radboud University Medical Center Nijmegen, Nijmegen, The Netherlands; Neuroepidemiology Section, Intramural Research Program, National Institute on Aging, National Institutes of Health, Bethesda, MD USA; Pathology (Neuropathology) and Neurological Sciences, Rush Alzheimer’s Disease Center, Rush University Medical Center, Chicago, IL USA; Department of Neurology, Stroke Division, Brigham and Women’s Hospital, Boston, MA USA; Division of Research, Kaiser Permanente Northern California, Oakland, CA USA; McKnight Brain Institute, Division of Cognitive Disorders, Neurology, Public Health Sciences and Neuroscience, University of Miami, Miami, FL USA; Neurology, Henry Ford Hospital, Detroit, MI USA

## Abstract

As the human population continues to age, an increasing number of people will exhibit significant deficits in cognitive function and dementia. It is now recognized that cerebrovascular, metabolic and neurodegenerative diseases all play major roles in the evolution of cognitive impairment and dementia. Thus with our more recent recognition of these relationships and our need to understand and more positively impact on this world health problem, “The Leo and Anne Albert Charitable Trust” (Gene Pranzo, Trustee with significant support from Susan Brogan, Meeting Planner) provided generous support for this inaugural international workshop that was held from April 13–16, 2015 at the beautiful Ritz Carlton Golf Resort in North Naples, Florida. Researchers from SUNY Downstate Medical Center, Brooklyn, NY organized the event by selecting the present group of translationally inclined preclinical, clinical and population scientists focused on cerebrovascular disease (CVD) risk and its progression to vascular cognitive impairment (VCI) and dementia. Participants at the workshop addressed important issues related to aging, cognition and dementia by: (1) sharing new data, information and perspectives that intersect vascular, metabolic and neurodegenerative diseases, (2) discussing gaps in translating population risk, clinical and preclinical information to the progression of cognitive loss, and (3) debating new approaches and methods to fill these gaps that can translate into future therapeutic interventions. Participants agreed on topics for group discussion prior to the meeting and focused on specific translational goals that included promoting better understanding of dementia mechanisms, the identification of potential therapeutic targets for intervention, and discussed/debated the potential utility of diagnostic/prognostic markers. Below summarizes the new data-presentations, concepts, novel directions and specific discussion topics addressed by this international translational team at our “*First Leo and Anne Albert Charitable Trust ‘Think Tank’ VCI workshop”.*

## Workshop participant presentations

### Philip Gorelick (Michigan State University, USA) started the meeting by commenting on vascular risks for cognitive impairment with a historical perspective in order to help us see our way forward

It has long been believed that traditional cardiovascular risk factors for stroke are risk factors for vascular forms of cognitive impairment, as well as late-onset dementias and Alzheimer’s disease (AD). Epidemiologic observational studies have supported this, and over time it has been shown that traditional cardiovascular risk factors are also risk factors for Alzheimer’s disease (AD). At this time this finding was largely unexpected. Reaching this knowledge plateau was delayed in part as a major operational definition of AD in the 1980s that essentially excluded the presence of traditional CVD risks [1]. Over time neuropathological-clinical translational studies caught up with hypothesis generation and showed that many cognitively impaired persons in later life had vascular lesion neuropathology that might also explain existing cognitive impairment [2–4]. The discovery that shared cardiovascular risks and mechanistic pathways were linked in both vascular and “neurodegenerative” disorders resulting in later life dementia supported this more contemporary thinking. This has led us to better define specific upstream pathophysiologic mechanisms contributing to neurovascular changes contributing to the cognitive dysfunction phenotype. We are encouraged by large scale epidemiological studies on incidence and prevalence of longitudinal cognitive function that show maintenance of cognitive vitality over time, which may be due at least in part to better control of or reduced cardiovascular risks. However, clinical trials of common cardiovascular risks such as hypertension, diabetes mellitus, and lipid disorders have not consistently shown clear concordance. Therefore, one must keep in mind that although the longitudinal epidemiological studies may suffer from confounding, they have the advantage of long-term follow-up which could begin as early as midlife. Also, cardiovascular preventive measures to reduce risk may need to be administered earlier in life and sustained over time for there to be a significant benefit at reducing the evolution of cognitive impairment to dementia. As we move forward in the field, there remain gaps in our understanding of molecular and cellular mechanisms and biomarkers for vascular causes of cognitive impairment and dementia. Many questions exist. For example: what are the molecular or cellular mechanisms of factors such as lipids, cell types related to innate and adaptive immunity, the blood brain barrier, blood flow, the role of traditional cardiovascular risks that are implicated in causation? Specific strategies that lead us to answers of important questions about vascular causes of cognitive disorders include: (1) development of a research network for the study of vascular mechanisms and risks for cognitive phenotypes as we age, (2) a scientific focus that includes basic pathophysiologic mechanisms and translational approaches, study of high resolution neuroimaging-clinical-neuropsychological-neuropathological outcomes, and development of multi-modal prevention and treatment studies, and (3) a lifecycle focus that includes provision of long-term studies including midlife and late life, as the benefits and adversities associated with prevention may result in different outcomes depending on different life stages. He emphasized that our mechanistic focus will need to remain upstream in order to intervene and halt downstream damage and dementia progression effectively.

### Deborah Gustafson (SUNY Downstate Medical Center, USA) suggested that published data support AD as a vascular disease

She summarized relevant epidemiological studies reporting that vascular risk factors, such as being overweight and obesity, hypertension, high blood levels of cholesterol, and type 2 diabetes mellitus, can increase risk for a clinical diagnosis of late-onset, sporadic AD [5]. The importance of CVD risk factors in AD has been consistently reported in the epidemiological literature for over 30 years, and global awareness increases. Vascular pathology in both brain and periphery is associated with AD, and new genetic findings enrich the evidence base for vascular factors in AD and involvement of peripheral metabolism in health of the brain [6]. Why are vascular factors associated with risk for a late life neurodegenerative disorder, such as AD? It was proposed that neurodegenerative events in AD, involving amyloid and tau, are vascular. The biology, systems and genes, as well as the neuropathology and imaging modalities used to diagnose the disorder, support this. Appreciating the breadth of what AD is explains and links the epidemiology, neuropathology, and clinical manifestation of this disorder of latest life–vascular AD. Many lines of evidence underscore vascular risk in AD, originating from neuropathological, imaging, mechanistic, genetic, clinical and epidemiologic studies. Interactions between brain and periphery, and the important role of the blood brain barrier, support the role of vascular factors. Accelerated progression of atrophy in AD measured using serial MRI is associated with vascular risk factors. Cerebral hypoperfusion, resulting from atherosclerosis and hypertension, increases AD risk. As such AD augments itself via multiple mechanisms. This is not to exclude other vascular and/or neurodegenerative events occurring alongside AD [7–9]. Observations of multiple ‘causes’ of AD due to neuritic amyloid plaques, Aβ fragments, neurofibrillary tangles, amyloid angiopathy, leukoariosis, atrophy, decreases in synaptic plasticity, altered dendritic spines, as well as apoptotic events could also point to a common underlying vascular pathology with heterogeneous manifestation due to neurovascular coupling. From clinical and epidemiological realms, vascular AD explains AD progression with corresponding changing symptom profiles, and even changing diagnoses over time. It is common in clinical and epidemiological studies for an individual to initially present with mild cognitive impairment, and be ‘normal’ at the next visit; or for a diagnosis of ‘*AD plus stroke*’, to become ‘*probable AD*’ over time, due to the slow progressive course of disease. The contribution of stroke in vascular AD is an obvious accelerator of dementia processes, and follows earlier Nun study [2] observations. Vascular AD may explain heterogeneous biomarker profiles (cerebral spinal fluid; CSF and imaging) and relative lack of specificity for those with clinical AD. From the epidemiology, vascular AD also explains observations of different vascular risk factor profiles for AD across populations with different magnitudes of effect, yet clinical AD occurring in all. This is also a product of differential susceptibility due to environment, lifestyle, and genetic background, as well as differences in specific times of life studied. Vascular AD explains the often transient or treatable declines in cognition observed in type 2 diabetes, human immunodeficiency virus/acquired immunodeficiency syndrome (HIV/AIDS), cancer, and other medical conditions, that have been said to ‘*look like*’ AD, but due to their transience and/or altered imaging or CSF biomarker profiles may be ‘*something else*’. Thus, AD is CVD based related to two fundamental elements of brain biology: APP and neurovascular coupling. These two elements align to illustrate why an amyloid-based model of AD is vascular based, and to support neurovascular coupling as an underlying mechanism for AD. This evidence advances the forum for scientific discourse related to AD neurodegenerative processes to include CVD and its role(s) in dementia. She concluded by emphasizing again that ‘*AD is Vascular AD*’.

### Geert Jan Biessels (Brain Center Rudolf Magnus, The Netherlands) described advances in detecting dementia vascular burden using higher resolution imaging approaches that extend significantly beyond conventional lesion detection

Previous autopsy studies have identified vascular pathology in the majority of patients with dementia and also in those with a clinical diagnosis of AD. Detection of this vascular pathology during life mainly relies on magnetic resonance imaging (MRI). The typical MRI manifestations of vascular pathology in the brain—in particular parenchymal lesions linked to small vessel disease—are common and are clearly associated with dementia risk. In fact, both in research and clinical practice these MR lesions have almost become our conceptual equivalent of small vessel disease. However, small vessel disease lesions represent an end-stage, are not sufficiently specific to understand disease processes and actually do not reflect abnormalities of the small vessels themselves. Moreover, small vessel disease lesion-burden alone often relates poorly to cognition in individual patients. Here he highlighted novel approaches to zoom in on novel imaging manifestations of small vessel disease and on some of the vascular abnormalities that may actually cause small vessel disease lesions, using high field 7T MRI. He showed how 7T MRI now enables us to study abnormalities in intracranial vessel structure and function with unprecedented detail [10–12]. These novel techniques are now being implemented in further studies to unravel the aetiology of small vessel disease. Moreover, I demonstrated that cortical microinfarcts, a very common form of ischemic brain injury related to cognitive decline and dementia, can now be detected with 7T in vivo [13]. The delineation of the imaging features of microinfarcts on 7T MRI now also helps to recognize them on 3T MRI, allowing a more rapid implementation of microinfarcts as a novel marker in vascular cognitive impairment research. We have already seen in a collaborative project with the team of Dr Chen from Singapore that these lesions do have specific cognitive correlates in a memory clinic population [14]. He also discussed work on MRI and MR-post processing approaches that better reflect the functional burden of small vessel diseases, also in individual patients. The potential of so-called ‘lesion symptom mapping’ was highlighted. This takes location of small vessel disease lesions, also in relation to main white matter tracts into account [15]. In addition, the potential of advanced brain network analyses with diffusion tensor imaging in the context of vascular cognitive impairment was discussed [16]. These developments in MRI technology and image analysis have great potential to improve our understanding of the aetiology of the vascular burden in dementia, will help to make a more accurate diagnosis, and will ultimately contribute to the development of targeted treatment.

### Howard Crystal (SUNY Downstate Medical Center, USA) presented data from his collaborative work on Vascular Health and Cognition in Women with HIV

Highly active antiviral therapy (HAART) has been widely available in the United States for nearly 20 years. Most studies report that with HAART the prevalence of HIV-associated dementia is markedly decreased, but milder forms of cognitive impairment remain common with prevalence estimates as high as 50 % [17]. As vascular health is associated with preserved cognition [18], and because both HIV and HAART can be vasculopathic, he hypothesized that vascular health would account for much of the variance in cognitive scores, and that vascular dysfunction would be associated with changes on volumetric MRI. Women from the Brooklyn site of the women’s interagency HIV study had measures of both large vessels (e.g., carotid intima media thickness and extracranial vessel velocities) and small vessels (e.g., pulsatility index of intracranial arteries and metrics from retinal photographs). 3T MRI with diffusion tensor imaging (DTI) sequences were obtained at New York University and analyzed by members of the Stebbins laboratory at Rush. Neuropsychological batteries were administered every 2 years, and summary measures of HAART-use over up to 20 years were obtained. High quality MRI and retinal photographs were available on 34 HIV-infected and 21 HIV-uninfected, age-matched women. He reported the effects of HIV-infection and HAART on vascular health, cognition, and MRI; the effects of vascular health on cognition, and the associations between vascular health and MRI volumetrics, and between MRI and cognition. Initial analyses indicated that increasing arterial dimension was associated with higher volumes, but increasing venous dimension was associated with lower volumes. According, he explored a novel measure—fractal dimension ratio (i.e., the arterial fractal dimension divided by venous fractal dimension)—as a summary measure. Significant partial correlation coefficients between fractal dimension ratio and 4 of the 6 volume measures and 4 of the 5 selected white matter integrity measures. In contrast the partial correlations between arterial or venous fractal dimension and the MRI measures were rarely significant. He suggested that retinal fractal dimension may be a surrogate marker of brain small vessel disease.

### Alison E. Baird (SUNY Downstate Medical Center, USA) discussed recent and near-future advances in nucleic acid-based diagnosis of stroke that also have potential for use in VCI

Here the rationale for the use of blood-based nucleic acid biomarkers to advance stroke diagnosis, treatment and prognosis determination was provided [19, 20]. She described how stroke is a leading cause of death and disability in adults, but at present, treatment for ischemic stroke reaches only a small percentage of patients. This is because of the very short time window for treatment and the time-consuming evaluation involved. She highlighted the intense efforts that are underway to find novel approaches to expedite stroke diagnosis and treatment demonstrating the strong rationale for the use of blood-based nucleic acid biomarkers to advance stroke diagnosis, as well as prognosis and prediction of VCI [21]. It was also reported that so far, no blood marker has emerged for clinical application in acute stroke management during the time window for treatment. Messenger ribonucleic acid markers have been identified in genomic profiling of circulating leukocytes that show promise for stroke detection and classification but technological issues are hindering the application of these microarray-derived results [22–24]. The sensitivity and specificity of transcript panels for ischemic stroke detection require further investigation, along with the time course of expression changes and which leukocyte subsets are the predominant sources of the gene expression changes. She then described the efforts currently underway to develop novel platforms for stroke diagnosis to meet the criteria for effective treatment within the narrow time window mandated by the FDA (<3 h) and to expedite further research studies into the application of the transcript panels for stroke diagnosis. Her collaborators, Dr. Soper and his group, are developing a lab-on-a-chip technology that provides the quantitative measurement of multiple transcripts in a turn-around-time of 15–22 min. The processing steps involve cell counting, cell subset, ribonucleic acid extraction and single pair fluorescence resonance energy transfer readout to provide an absolute copy number of as many as 30 transcripts. The technology is independent of polymerase chain reactions and uses chips the size of a quarter that cost a few pennies. Meanwhile her group has been performing clinical studies in tandem to address issues of transcript quantitation, transcript optimization, determination of expected expression levels in stroke and control condition and determination of which leukocyte subsets to employ [25]. This work is showing how nucleic acid blood-based biomarkers coupled with new analytical tools and can serve as an attractive platform for managing stroke-related diseases in the emergency and outpatient clinical settings that might also be extended to VCI [26].

### Mateusz G. Adamski (Jagiellonian University, Poland) described the potential for circulating blood cells as potential biomarkers and active players in the development of VCI

VCI is recognized to be associated with the development of vascular diseases and subsequent compromise of the blood brain barrier (BBB) permeability. New roles of circulating blood immune cells, representing both innate and adaptive responses, have been recently identified to play a major role in the pathomechanism of two major VCI risk factors: stroke and hypertension. These new findings were very intriguing as they challenged the notion that due to anatomical and physiological limitations, full immune response cannot develop in central nervous system. These limitations included: early formation and development of BBB, lack of cerebral lymphatic vessels, inefficiency of microglia and astrocytes for antigen presentation to T cell lymphocytes, high rate of apoptosis in cells crossing BBB, assumption of the passive function of neurons and short time for the adaptive immune response to develop especially in acute stroke. Thus, selected results from clinical and experimental studies were presented to show that cells representing both innate and adaptive immune systems as well as bridge cells—having features of both adaptive and innate immunity—contribute to the development of VCI risk. First, the results on the role of invariant natural killer T cells and granulocytes, both representatives of innate immune system, in experimental and clinical stroke were presented. Immunomodulation of invariant natural killer T cells was shown to be protective in mice stroke model [27]. Expression of 3-gene cluster in CD15 granulocyte in acute ischemic stroke patients was shown to be highly sensitive (89 %) and specific (67 %) in classifying stroke (unpublished). Second, the role of several populations of CD4 T cells and B cells, representatives of adaptive immune system, from clinical and experimental stroke studies was presented. Stroke clinical studies showed differences in function and frequencies of several subsets of circulating T- [28, 29] and B-lymphocytes [30]. Experimental stroke studies showed that T regulatory lymphocytes induce endothelial dysfunction and increase infarct size [31] and that depletion of IL-21 producing T cells improves stroke outcome [32]. Third, the role of natural killer T cells and γδT cells, representatives of bridge cell populations, was presented in stroke and hypertension. Clinical stroke induced changes in absolute numbers of both natural killer T cells and γδT cells, with hypertension being the strongest factor causing γδT cell reduction [33]. Experimental study showed that subsequent to stroke, γδT cells infiltrate brain and secret IL-17A—detrimental for ischemic brain [34]. Other participants at this Inaugural workshop highlighted the importance of VCI in the development of dementia and stressed the need to identify underlying mechanisms responsible for the disease. Therefore, circulating blood cells can be involved in diverse and pathological vascular mechanisms and are promising potential target opportunities as biomarkers and active players in the context of VCI.

### Clotilde Balucani (SUNY Downstate Medical Center, USA) discussed her collaborative research exploring cognitive performance and cerebral hemodynamics in bilateral asymptomatic carotid stenosis

The work was conducted with Mauro Silvestrini, MD (Neurological Clinic, Marche Polytechnic University, Ancona, Italy). Few studies have highlighted the association between severe carotid atherosclerosis markers (i.e. increase in carotid intima–media thickness and carotid plaques) and an increased risk of cognitive impairment, presence of silent cerebral infarcts, white matter lesions, and reduced parietal grey matter. Here she evaluated cognitive performance in subjects with bilateral asymptomatic carotid stenosis compared to subjects with unilateral asymptomatic carotid stenosis and to subjects with no carotid stenosis and to explore the relationship between cognitive performance and cerebral hemodynamics status. Further longitudinal cognitive and hemodynamic performance was measured in subjects with bilateral and unilateral asymptomatic carotid stenosis. Cerebral hemodynamics status was assessed using the transcranial Doppler-based breath-holding index test. The neuropsychological investigation included tests for exploring the left and right brain functions. In the longitudinal cohort the cognitive status was evaluated using the Mini-Mental State Examination at baseline and at 3 years. The study included 333 patients. Subjects with bilateral and unilateral asymptomatic carotid stenosis were significantly more likely to have cognitive dysfunction compared to subjects with no carotid stenosis. Further, there was a statistically significant association between cognitive dysfunction and cerebral hemodynamic impairment resulting from the carotid stenosis. In the group of 159 patients followed-up at 3 years, the risk of cognitive impairment increased progressively from patients with bilaterally normal to those with a bilaterally abnormal breath-holding index. These findings support the contributing role of carotid stenosis in the development of cognitive impairment. The evaluation of the hemodynamic status, besides providing insights about the possible mechanism behind the development cognitive dysfunction in otherwise asymptomatic carotid disease, may be of help for the individuation of subjects deserving earlier and more aggressive treatments.

### Rachel Whitmer (Kaiser Permanente Northern California, USA) discussed diabetes mellitus and dementia experimental model work designed to better understanding vascular contributions to brain aging

Although vascular risk factors are associated with increased risk of dementia and markers of accelerated brain aging, the mechanisms and timing of influence are not clearly delineated. Diabetes mellitus, a metabolic condition with numerous vascular sequelae is rapidly increasing in incidence worldwide. While it is established that those with type 2 diabetes have twice the risk of dementia and cognitive impairment, less is known regarding how the characteristics of this disease impact brain health. Specifically, the role of diabetes treatment, micro/macrovascular complications and long-term glycemic control is little understood. Those with type 1 diabetes, a disease with onset typically in childhood or adolescence and with immediate insulin treatment, are now living to old age, whether these individuals are at higher risk of age-associated neurodegenerative disease has not been studied. We have been following over 20,000 elderly individuals with diabetes mellitus since 1994 in the Kaiser Permanente Northern California Diabetes Registry. Here recent findings on long-term glycemic control and vascular complications of diabetes on dementia risk were presented with a primary objective of identifying diabetes-associated characteristics which impact dementia and how this translates to the general population and better elucidation of potential vascular-brain mechanisms. A newly launched NIH funded Study of Longevity in Diabetes (SOLID) study is now following elderly patients with type 1 diabetes to ascertain the cognitive aging signature of this disease and to identify risk and protective factors for cognitive impairment in this population newly entering old age.

### Amanda Kiliaan (Donders Institute for Brain, Cognition and Behaviour, The Netherlands) presented on the impact of diet on cerebral circulation, neuronal integrity and cognition in mouse models for vascular risk factors in AD

The important role of diets and healthy lifestyle as preventative of vascular diseases is widely accepted. Recently, the Mediterranean diet (e.g., containing polyunsaturated fatty acids as important components) has been shown in several prospective world-wide studies to be inversely associated with CVD and to be a strong protective factor against hypertension, obesity, and AD [35–38]. Dietary lipids are beginning to be recognized for their direct actions on synaptic function and cognitive processes. However, diets high in saturated fat are becoming notorious for reducing molecular substrates that support cognitive processing and increasing the risk of neurological dysfunction in both humans and animals. Previous and new preliminary data in mice models of AD and vascular impairment show that diet (i.e., containing several components of the Mediterranean diet) is able to inhibit/reverse the early functional connectivity reduction and impairment in cerebral blood flow [39–41]. Both parameters are apparent already before cognitive decline. Thus, diet changes may provide preventive strategies in very early, non-symptomatic phases of AD. Recent focus is therefore on the early, asymptomatic phase of the disease especially on the cardiovascular-metabolic risk factors including atherosclerosis, hypertension, obesity and Type II Diabetes that can be modified by changes in diet lifestyle. In order to validate potential therapeutic targets several transgenic and non-transgenic animal models have been developed to elucidate the mechanistic aspects of AD. Mostly, transgenic rodent models over-expressing human amyloid β (Aβ) precursor protein (β-APP) and mutant forms of tau have become important tools to study the pathogenesis of AD [42], and to test new therapeutic agents. Nevertheless, none of the transgenic models of AD recapitulate fully all of the pathological features of the disease and they don’t take into account the effect of the most important aspects of human AD, namely risk factors in aging, CVD, and gender. It should be mentioned that sex is an important risk factor for both AD and stroke, as both conditions appear to affect women more than men [43, 44]. In contrast, male sex is considered a risk factor for mixed vascular-AD dementia [45]. The sex factor is often ignored when designing experimental studies investigating stroke-related vascular disorders and AD. So-called mixed dementia, with post-mortem pathological evidence for both cardiovascular disease and AD, is now thought to be much more common in the elderly than any of the ‘pure’ forms of dementia. Recent advances in imaging techniques now allow for the in vivo visualization of parameters such as brain volume and connectivity (MRI), gray and white matter integrity (MRI-DTI) and blood perfusion (MRI arterial spin labelling). In dementia and cardiovascular disease, imaging has provided valuable differential diagnostic information, for example demonstrating the atrophy patterns and Aβ deposits typical for AD [46] and the lesions and network reorganizations associated with cardiovascular disease [47]. However, none of these animal studies have examined neuroimaging parameters, although they have mainly focussed on elucidating the bidirectional interaction between β-APP/Aβ and ischemic injury and the potential role of inflammation. As advanced neuroimaging techniques are now available for small animals such as mice [48, 49], it is feasible to gain more insight into AD pathology and the role of cerebrovascular disorders. There is a significant concern that most animal models for AD are showing translational failure and that improved translational models using translationally-validated diagnostic tools should be developed and utilized.

### Herman Moreno (SUNY Downstate Medical Center, USA) described the divergent pathologies Tau- and Aβ-42 have on the functioning of entorhinal—hippocampal circuits

Here he used the electrophysiological measurements in hippocampal slices from AD mouse models. The entorhinal cortex is the gateway into the hippocampal circuit and is affected early in AD. AD itself is defined by a co-occurrence of tau and amyloid-related pathologies. How the co-occurrence of these pathologies in the entorhinal cortex affects the electrophysiological profile of the hippocampal circuit remains unknown. He answered this question by performing detailed electrophysiological analyses of the circuit in three mouse models that selectively express in the entorhinal cortex the genetically-induced disease-causing mutations in mice expressing mutant β-APP (to produce increased oligomeric Aβ-42 peptides as in AD) or in human Tau, or both mutant β-APP and human Tau. Our findings confirm that β-APP and Tau expression produce contrasting electrophysiological effects. Entorhinal cortex-β-APP expression caused spontaneous network hyperexcitability, in the superficial layers of the entorhinal cortex. Additionally, pyramidal cells of the subiculum, which are monosynaptically connected to entorhinal cortex, showed decreased miniature excitatory postsynaptic current amplitude, which may represent a synaptic homeostatic response to increased neuronal activity at the entorhinal cortex level of mice expressing β-APP. Human Tau expression was associated with an effective decrease in induced synchronous discharges, observed in the entorhinal cortex-hippocampal circuit and no significant changes in subicular neuronal miniature excitatory postsynaptic current amplitude. Entorhinal cortex-β-APP expression resulted in an increase in the paired-pulse facilitation normally seen in the entorhinal cortex-subiculum synapse, while entorhinal cortex—human Tau expression resulted in a decrease. In humans, amyloid pathology in the entorhinal cortex occurs on the background of Tau pathology, therefore oligomeric Aβ-42 peptides were applied in the entorhinal cortex-hippocampal slice and produced an acute increase in both spontaneous and evoked synaptic events in subicular neurons of normal wild-type mice. Human Tau-expressing mice did not respond to oligomeric Aβ-42 peptides. Next, mice expressing mutations of both mutated β-APP and human Tau in the entorhinal cortex were studied. The electrophysiological profile in the mice co-expressing both mutant β-APP and human Tau shows that human Tau has a dominant effect, generally dampening the excitatory effects of β-APP. Thus, these data suggest that the occurrence of epileptiform activity in human AD may depend in the ratio of mutant β-APP and resultant oligomeric Aβ-42-to Tau-induced pathologies. He is now extending this work to investigate the changes that can be produced by acute stroke in the background of β-APP- Aβ and Tau pathology, and is also characterizing the functional presynaptic changes produced by Tau pathology under these conditions.

### Zheng Gang Zhang (Henry Ford Hospital, USA) presented new data on how stroke exacerbates neurovascular and white matter damage in diabetic rats

Diabetes is a common metabolic disease in the aged population, which leads to an increase of stroke incidence and slower neurological recovery. His recent data demonstrates that middle age rats with type II diabetes exhibit spatial learning deficits. Immunostaining analysis showed the presence of microvascular leakage and loss of axons and mature oligodendrocytes in the hippocampus. Focal cerebral ischemia exacerbated neurological outcomes and learning deficits during stroke recovery in middle-age diabetic rats compared to age-matched ischemic rats without diabetes. Stroke increased vascular leakage and reduced oligodendrocytes and myelinated axons within the hippocampus of diabetic rats. However, stroke did not increase stroke-induced infarction in diabetic rats. In addition, he found that diabetes activates the toll-like receptor/IL-1R-associated kinase signaling pathway in cerebral endothelial cells and that blocking this signaling pathway with a tetrapeptide N-acetyl-seryl-aspartyl-lysyl-proline (AcSDKP) substantially reduced neurovascular and white matter damage and improved neurological outcome including learning deficits after stroke.

### Ken Arai (Harvard Medical School, USA) discussed the roles of oligodendrocyte precursor cells in cerebral white matter under physiological and pathophysiological conditions

White matter damage is a clinically important aspect of several CNS diseases including stroke or vascular dementia. For example, increased white matter lesions are well known to be negatively correlated with cognitive function [50]. White matter primarily consists of axonal bundles ensheathed with myelin by mature oligodendrocytes, and plays an important role in passing signals between different areas of gray matter. However, compared to the mechanisms of neuronal injury in gray matter, white matter pathophysiology such as oligodendrocyte death and myelin damage remains relatively understudied and poorly understood. During development, oligodendrocyte precursor cells migrate from the ventricular zone to their destination and then differentiate into mature oligodendrocytes to form myelin sheaths. Although myelinated tracts are formed early in life, renewal of myelin and oligodendrocyte continues throughout most of the adult life. Residual oligodendrocyte precursor cells may also play an important role in the endogenous mechanisms of white matter repair and renewal after white matter damage [51, 52]. Here he discussed oligodendrocyte precursor cell functioning in cerebral white matter under both physiological and pathophysiological conditions. New data indicates that oligodendrocyte precursor cells are important for white matter remodeling and repair in the mouse model of prolonged cerebral forebrain hypoperfusion. Notably, compensative responses in these precursor cells were dampened by ageing. Compared to young mice (2–3 months old), a pro-survival “cyclic adenosine monophosphate response element-binding protein” signaling was suppressed in middle-aged mice (8–10 months old), which disturbed precursor cell differentiation (i.e. reduced oligodendrogenesis) after white matter injury. Since activating this pro-survival signaling by cilostazol was supportive for white matter function against hypoxic stress even in middle-aged mice, the cyclic adenosine monophosphate response element-binding protein signaling would be a potential therapeutic target for white matter-related diseases [53]. In addition, another important aspect of oligodendrocyte precursor cells was discussed. These precursor cells might not be “mere progenitors” for mature oligodendrocytes. Rather, these cells may communicate closely with neighboring cells to actively maintain white matter homeostasis. Using pharmacological and genetic approaches he demonstrated that precursor cell-derived factors regulate the integrity of the blood brain barrier in cerebral white matter. For example, under normal conditions, precussor cells secret TGFβ that strengthens blood brain barrier integrity [54]. However, under pathological conditions, these cells can “attack” or damage the blood brain barrier by secreting matrix metalloproteinases, which eventually leads to white matter/myelin dysfunction [55]. Although oligodendrocyte precursor cells are one of the major cell types in adult white matter, their roles are still mostly unknown. Therefore, a deeper understanding of OPC roles after white matter damage can be expected to lead us to novel therapeutic approaches for CVD and VCI.

### David Cechetto (Western University, Canada) provided a detailed discussion of pathological mechanisms and the therapeutic potential of co-morbid models that use combined vascular plus AD risk factors

Clearly, CVD and vascular risk factors exacerbate cognitive decline in AD. Animal models have now provided considerable insight into the interaction of vascular risk factors such as stroke or diabetes and high levels of amyloid in the brain. Here vascular risk models included the use of small striatal strokes in rats consuming high fat and caloric diets. In addition, these risks were combined in AD models that involved the use of transgenic AD mice or rats that received injections of Aβ into the cerebral lateral ventricles. Outcomes measured include cognitive deficits, AD-like pathology, neuroinflammation, oxidative stress, measures of gap junction proteins, membrane raft proteins and glanglioside levels, brain infarct size, hippocampal progenitor cell proliferation and migration, neurovascular unit disruption, MRI imaging of structural changes, and cerebral blood flow changes using CT imaging. Typically, these outcome measures exhibit an enhanced pathological change when cerebrovascular manipulation or vascular risk factors are combined in AD models or with cerebral ventricular Aβ. Some of these changes in outcome occur early in the cascade of events (i.e., precede major neurodegenerative changes), and thus can be examined as possible therapeutic targets for intervention to prevent subsequent pathological changes and cognitive decline.

### Frank C. Barone (SUNY Downstate Medical Center) described a translational rat model of VCI that combines the hypertension and carotid artery stenosis risk factors known to produce VCI in man

VCI is associated with CVD and involves reduced forebrain perfusion. Symptoms and signs of VCI include deficits in executive function, working memory and significant problems in gait/balance. Our current limited understanding of VCI mechanisms emphasizes our need for animal models that represent these factors, symptoms and pathological changes exhibited by VCI in man [56–59]. Here hypertension (i.e., to mimic small vessel disease that can significantly impair cognition in man) and reduced forebrain hypoperfusion (i.e., using bilateral carotid artery stenosis to mimic the consequences of carotid atherosclerosis) were incorporated together in order to create a relevant model for future VCI translational research. Bilateral common carotid artery stenosis surgery (i.e., guided by reduced forebrain microvascular perfusion) or sham surgery was performed in spontaneously hypertensive rats (SHR). SHR were evaluated on a series of neurological (gait, balance and sensory-motor) and cognitive (active place avoidance; APA) tests. Several separate experiments have been conducted to establish reliability and validation of the developed model. Histological measurements of corpus callosum fiber tract myelin loss, microvascular changes and increased microglia and astrocytes were measured to investigate if brain changes were similar to those observed in human VCI. SHR with stenosis surgery exhibited a persistent decrease in forebrain perfusion resulting in significant deficits in APA learning and gait/balance occurring within 3 weeks and persisting for over >3 months. Stenosis SHR do not learn to avoid entry into a shocked location on a moving APA arena, while similar total path/movement is traveled by both groups in the test environment. Stenosis SHR deficits were not related to differences in general motor activity, sensory-motor deficits, peripheral cardiovascular measures or body weight. In the corpus callosum, stenosis produced significant myelin loss and increases astrocyte and microglia activation and proliferation. Thus, carotid stenosis in the hypertensive rat results in cognitive and behavioral changes that mimic human VCI [48, 60] and as seen in other related rat models [61, 62]. The APA learning paradigm requires complex cognitive control and is a sensitive detector of VCI-like cognitive deficits. Thus, this “carotid stenosis plus hypertension model” combines important cerebrovascular risk factors that mimic known contributing conditions and produces symptoms analogous to human VCI. It is expected to be an important tool for preclinical studies that can probe VCI pathophysiology, mechanisms and intervention. Plans are to use the model to investigate therapeutic interventions that reduce microvascular and brain inflammation and myelin loss, promote restorative changes and to evaluate relevant biomarkers. Ongoing work includes MRI and positron emission tomography imaging and circulating molecular and cellular biomarker research.

### Julie Schneider (Rush University Medical Center, USA) discussed how CVD is an important determinant of AD dementia and that impairment in multiple cognitive domains occurs in aging

Although CVD pathology is very common in aging its associations with Alzheimer’s disease (AD) dementia and with specific cognitive domains is not completely understood. She investigated the role of small vessel (arteriolosclerosis and cerebral amyloid angiopathy) and large vessel (atherosclerosis) diseases and also micro and macro brain infarctions that occurred in AD dementia patients including several domains of their cognitive impairment. Over 1000 older persons without dementia from 2 longitudinal clinical-pathologic studies of aging, the Rush Memory and Aging Project and the Religious Orders Study, all undergo annual clinical evaluations. After death, a clinician reviews all data, blinded to neuropathologic information to establish a final clinical diagnosis. Neuropathologic examinations were also blinded to clinical data and providee standardized measures of each CVD pathology. The association of each type of CVD with the odds of AD dementia and cognitive domains was examined using logistic and linear regression models. Significant CVD pathology is a very common occurrence in the brains of older persons (i.e., present in >75 % of participants and commonly co-occurs with AD pathology). However, only cerebral amyloid angiopathy (i.e., but no other CVD pathology) was correlated with AD pathology. In analyses that were adjusted for age-related neuropathologies, arteriolosclerosis, atherosclerosis, cerebral amyloid angiopathy, and infarcts were all independently associated with an increased odds of AD dementia. Furthermore, each of these pathologies was associated with impairment in multiple cognitive domains including not only perceptual speed but also episodic and semantic memory (language). The deleterious effects of CVD were independent of AD pathology and were present in persons both with and without dementia. Thus, these data analyses indicate that CVD is an important determinant of AD dementia and relates to significant impairment in multiple cognitive systems in old age.

### Adam M. Brickman (Columbia University, USA) focused on the harbingers of AD, including risk factors, biomarkers, and white matter hyperintensities

Prevailing hypotheses on the pathogenesis of AD include a cascade of biological events that emphasizes abnormal β-amyloid processing and tau-related neuronal and synaptic dysfunction that lead to dementia. However, despite fairly consistent observations demonstrating the relationship of vascular risk factors and frank vascular disease to AD, vascular factors have not been incorporated formally into the proposed theoretical model of AD pathogenesis or newly proposed research criteria for AD and its antecedent conditions. The gradual accumulation of vascular risk factors are manifested in the brain as cerebrovascular small vessel disease, best visualized on T2-weighted MRI scans as white matter hyperintensity. He has systematically investigated the contribution of white matter hyperintensity to the clinical presentation of AD and tested the extent to which these hyperintensities interact with other markers of AD pathology. His research shows that these hyperintensities reflect pathology that is independent of AD pathology, conferring additive risk or contribute to symptom presentation. His work also shows that hyperintensities may interact more directly with AD pathology, conferring a synergistic effect on clinical outcomes.

### Lenore Launer (National Institute on Aging, NIH, USA) described the importance of microvascular disease brain injury in AD and dementia

Microvascular disease is systemic, increases with age, is accelerated by vascular risk factors, and causes clinically detectable damage to multiple organs such as the retina, kidney, heart and brain. Microvascular disease in the brain refers to the narrowing and occlusion of tiny arteries or arterioles that penetrate the brain cortex, reaching the underlying structures of the white and gray matter. Some microvascular lesions, such as diffuse white matter changes and focal microbleeds, can be seen on standard MRI. However, other lesions, such as microinfarcts, are below the limits of resolution of today’s clinical neuroimaging methods and can only be seen on microscopic examination of brain tissue. Recently several autopsy studies, including the Honolulu-Asia Aging Autopsy Study, have identified microinfarcts as pervasive and very prevalent in the brains of older demented and non-demented persons [63]. She emphasized that there is nothing small about the consequences of microvacular disease. Cumulatively, these microvascular lesions represent major contributors to age-related cognitive decline and dementia. She used longitudinal data (unpublished) from the Age, Gene/Environment susceptibility (AGES)-Reykjavik study [64] and demonstrated that the rates of dementia and its subtypes, AD and vascular dementia, were nearly doubled in the 20 % of participants who had evidence on MRI of new microbleeds. She described many important directions for future studies that could move the field towards new approaches for preventing or treating microvascular disease-related neurologic dysfunction including: developing standard neuropathological lesion measurement protocols, validating novel MRI methods to detect/measure microinfarcts, investigating the genetic and environmental risk factors leading to cerebral microvascular disease; developing tools to screen for possible microvascular disease and its consequences, and to unravel the potential mechanisms of interactions with AD lesions.

### Clinton Wright (University of Miami, USA) described the phenotypes of subclinical cerebrovascular damage and age-related cognitive outcomes learned from investigating a diverse urban population

We know that midlife modifiable risk factors for stroke and vascular disease are potent predictors of cognitive decline in late life, however, many intervening factors confound our understanding of why this occurs. Specifics on the biology of aging, cellular neurodegenerative processes, and vascular damage itself are poorly understood. Questions regarding the quantity, quality, and location of vascular injury required to take away normal cognition and replace it with vascular cognitive impairment are now only beginning to be answered. Multiple disciplines and scopes of understanding that range from the infinitesimal molecular processes of cell biology to big data and social networks will be needed to clarify many of these relationships. Population‐based studies provide insights into these complexities and his presentation highlighted examples of how structural imaging of brain changes can help us understand the different phenotypes. In addition, he discussed how commonly accepted markers of subclinical brain injury relate to cognitive outcomes.

### Farzaneh Sorond (Brigham and Women’s Hospital, USA) described ways to provide early detection of dementia using the neurovascular unit response as a biomarker for vascular functioning

CVD is an important cause of cognitive impairment and dementia. Intracranial small vessel disease rather than large strategic cortical lesions appear to be the major factor in the clinical expression of age related motor and cognitive decline. Brain parenchymal changes such as cerebral white matter hyperintensities, lacunar strokes and cerebral microbleeds have been identified as surrogate radiographic manifestations of small vessel disease. However, current evidence suggests that once established, radiographic small vessel disease is irreversible. Therefore, any treatment has to be directed at the vascular changes that precede radiographic small vessel disease. Yet, very few studies have examined pre-clinical cerebral vascular measures and their relationship to clinical and radiographic outcomes of cerebral small vessel disease. As the tools to measure cerebral small vessel function become increasingly more sophisticated we are now well poised to advance our understanding of the physiological changes underlying cerebral small vessel disease by directly studying cerebral small vessel function. The introduction of transcranial Doppler to measure cerebral blood flow velocity provided a powerful tool for non-invasive assessment of cerebral vascular responses to various physiological challenges such as motor or cognitive activation, or change in blood pressure and end-tidal carbon dioxide which we know are regulated at the level of arterioles or resistance vessels of the brain. Since the mechanisms underlying each of these vascular responses are likely different, they have been traditionally distinguished by different labels as follows: (a) Changes in cerebral blood flow velocity in response to sudden changes in blood pressure is referred to as “*dynamic cerebral autoregulation”*. (b) Changes in cerebral blood flow velocity in response to changes in end-tidal CO_2_ are referred to as “*cerebral vasoreactivity”*. (c) Changes in cerebral blood flow velocity in response to a motor or cognitive task is referred to as “*neurovascular coupling or functional hyperemia”*. In addition to these three functional measures, resting cerebral blood velocity waveform can be also used to calculate a “*pulsatility index”* which is a measure of cerebrovascular compliance. Using these approaches transcranial Doppler can provide continuous non-invasive beat-to-beat measurement of the cerebral blood flow velocity in the basal cerebral arteries with a high temporal resolution and has become the most commonly utilized tool to study cerebrovascular function in humans. A number of studies have shown that these vascular biomarkers measured by the transcranial Doppler are associated with radiographic verified small vessel disease. More recently, we and others have shown that these vascular measures are also associated with deficits in motor and cognitive function. Using transcranial Doppler cerebral vascular measures is expected to allow us to shift current practice away from surrogate late stage brain parenchymal MRI measures to these vascular biomarker measurements which directly measure cerebral vascular functioning at an early time when one may be able to intervene to prevent irreversible neuronal injury.

## Team selected discussion topics

In addition to individual participant presentations, several pre-selected discussion topics were addressed by this translational team. Discussion by the team of these topics is briefly summarized below. In a “*Biomarkers and Disease Monitoring”* session the need for markers of VCI was emphasized. The transcranial Doppler measures were considered to be important tools for future validation. Markers of axonal and/or myelin injury (e.g., MRI diffusion tensor imaging) that can assess progression or regression of changes related to VCI need further validation. New biomarker discovery and development is required to help us better identify those at risk and/or those with prodromal dementias (e.g., from blood and/or CSF). In the future these new markers may tell us about the diagnosis, prognosis, and underlying biology of vascular cognitive impairment. Early initiation and long-term epidemiologic studies continue to be required in order to better understand the etiologies affecting late life. Clearly, epidemiologic studies of dementia progression are now required. Projecting to futuristic clinical trials, it was suggested that cognitive preservation could be measured by molecular and cellular real-time monitoring connected to vascular cognitive centers that are associated with robust dementia networks.

In a “*Molecular and Cellular Disease Mechanisms”* session the beneficial vs. deleterious roles of glial cells after brain injury and neurodegeneration was highlighted. Basic work now needs to extend our understanding of the “bad” and “good” glial cell effects that can impact future effective therapeutic strategies. Cerebral endothelial cells and neighboring cells comprising the perivascular niche, provides a specific microenvironment for stem or progenitor cells to retain their multi-lineage potential and self-renewal capacity. It is felt that understanding the mechanisms of cell–cell interactions in the perivascular region would be important for developing regenerative therapies. We need to examine the dysfunction of cell–cell interactions under diseased conditions and to preserve the normal interactions and induce restorative changes after brain injury. What are the critical mechanisms that link risk factors (e.g., stroke, hypertension, atherosclerosis and Type 2 Diabetes) to increase brain vulnerability into a degenerating condition leading to dementia? Experimental models that demonstrate these interactions can be used to help study these mechanisms. Also, we require blood brain barrier experimental models and new approaches for clinical assessment of BBB function. It appears that the highest impact can be obtained by focusing on the initial stages in the process preceding dementia (e.g., early effects of co-morbidities on neuronal, glial, and cerebrovascular regulation and their relationship to high levels of brain amyloid, oxidative stress, matrix degrading enzymes and vascular permeability and axonal disruption). Questions were raised on expression of cognitive phenotypes for vascular damage in their most recognizable forms and how these phenotypes can be distinguished from normal aging and neurodegeneration. It was suggested that multiple therapies might be appropriate but that translational experimental approaches will be required to provide proof of principle for a combination of agents to intervene in this process which, of course, will require a more detailed understanding of combined Vascular-AD pathology.

Finally, in a session on “*Funding, Vascular Interventions and Animal Modeling”*, participants were interested in collaboration opportunities, in identifying creative ways to support and expand collaborative work together and in translating experimental and/or epidemiological findings into treatment. These continued ongoing discussions and collaborative work will be the subject of report updates and group discussions at our future workshops. It was considered important to develop mechanism based therapies with focused therapeutic approaches that really target the processes in the cerebral vasculature and downstream from there to subsequent effects that affect cognitive processing (i.e., not just risk factor management). It was emphasized that current animal models for AD show translational failure because they are cross-sectional and focus on single highly overexpressed factors. This differs significantly from human conditions. Animal models must take into account important aspects of human AD, such as aging, CVD, and gender. Aging is the strongest risk factor for AD, but we lack a valid animal model to study the effects of age on AD. In sporadic AD, the prevalence of vascular risk factors is high, and ignoring these vascular-disease factors when studying experimental AD has hampered translation. Gender strongly affects outcome in AD models, but this is commonly ignored and leads to erroneous interpretations, and thus to translation. Studying long-term, longitudinal changes are very important. Human AD is a chronic disorder with an asymptomatic phase of 20–30 years. Although longitudinal studies in humans are time consuming, animal models can speed this up significantly. Translational animal models will allow us to study life-time effects of aging, cardiovascular-metabolic changes, gender, and CVD on parameters known to be affected in human sporadic AD. For example, genetically and/or diet modified rodents can be utilized and subjected to in vivo brain administrations of Aβ followed by cognition testing and later brain atrophy and altered connectivity measurements, including post-mortem brain pathology and mechanistic histology-immunohistochemistry approaches that monitor inflammation, amyloid pathology, cellular and axonal injury and synaptic loss. The relevant imaging and fluid molecular data discussed at this workshop and by many others will need validation for use as biomarkers for future clinical use.

## ‘Think Tank’ VCI workshop: summary and future steps

Vascular disease was once considered the primary cause of age-related dementia [55–57]. Then this “vascular-centric’ thinking in AD had been ignored for a long period. Now we have returned back to this thinking based on much more data and a better understanding of the cardiovascular and metabolic contributions to cognitive loss with even greater focus on research emphasis to elucidate specific pathophysiologic mechanisms that contribute to dementia phenotypes and neuropathologic outcomes [65–69]. There have been major statements reviewed by global health groups [57, 58], diagnostic statements made regarding VCI [50, 70, 71] and other workshop/symposium summaries of this debate [72, 73]. Here in our ‘Think Tank’ workshop we represented expertise in neurology, neuropathology, epidemiology, neuroscience, preclinical science, and pharmacology, among other disciplines. Our focus was on CVD (including vascular and metabolic changes) and its involvement in the progression of cognitive impairment to dementia. Thus, we actively discussed or presented aspects of dementia in relationship to hypertension, atherosclerosis-carotid stenosis, diabetes, obesity, lipids, nutrition and chronic HIV infection. Imaging, cerebrovascular, behavioral and circulation biomarker development strategies that might be useful for diagnostic and prognostic purposes were also addressed. Epidemiological associations with vascular and metabolic risk factors were significant points of discussion. The interaction between cerebrovascular and metabolic-diet conditions and neurodegenerative disease was discussed in detail by this translational team. Given the success of this first workshop, The Leo and Anne Albert Charitable Trust has decided to support future meetings on a biannual basis in order to continue formal discussions/debates, address new knowledge/technology, continue and build collaborations including invitations to new participants. Based on the changing emphasis and new knowledge and our discussions at this meeting, we decided that our future ‘think tank’ workshops will refer to the spectrum of vascular and AD contributions to dementia as *Vascular*-*Alzheimer Spectrum (VAS) Disorders* [74]. All coauthors agreed to cooperate/collaborate together on VAS Disorders now and to bring together/consult experts in the future for our next workshop on *VAS Disorders* in 2017.

## Abbreviations

VCI: vascular cognitive impairment
CVD: cerebrovascular disease
AD: Alzheimer’s disease
CSF: cerebral spinal fluid
HIV: human immunodeficiency virus
AIDS: acquired immunodeficiency syndrome
MRI: magnetic resonance imaging
HAART: highly active antiviral therapy
DTI: diffusion tensor imaging
BBB: blood brain barrier
Aβ: amyloid β
β-APP: human amyloid β precursor protein
SHR: spontaneously hypertensive rats
APA: active place avoidance

## References

[1] McKhann G, Drachman D, Folstein M, Katzman R, Price D, Stadlan EM (1984). Clinical diagnosis of Alzheimer’s disease: report of the NINCDS-ADRDA work group under the auspices of Department of Health and Human Services Task Force on Alzheimer’s Disease. Neurology..

[2] Snowdon DA, Greiner LH, Mortimer JA, Riley KP, Greiner PA, Markesbery WR (1997). Brain infarction and the clinical expression of Alzheimer disease. The Nun study. JAMA.

[3] Neuropathology Group (2001). Medical Research Council Cognitive Function and Aging Study—Pathological correlates of late-onset dementia in a multicentre, community-based population in England and Wales—Neuropathology Group of the Medical Research Council Cognitive Function and Ageing Study (MRC CFAS). Lancet.

[4] Román GC, Tatemichi TK, Erkinjuntti T, Cummings JL, Masdeu JC, Garcia JH, Amaducci L, Orgogozo JM, Brun A, Hofman A (1993). Vascular dementia: diagnostic criteria for research studies. Report of the NINDS-AIREN International workshop. Neurology..

[5] Kiliaan AJ, Arnoldussen IA, Gustafson DR (2014). Adipokines: a link between obesity and dementia?. Lancet Neurol.

[6] Guerreiro RJ, Gustafson DR, Hardy J (2012). The genetic architecture of Alzheimer’s disease: beyond APP, PSENs and APOE. Neurobiol Aging.

[7] Zlokovic BV (2005). Neurovascular mechanisms of Alzheimer’s neurodegeneration. Trends Neurosci.

[8] Zlokovic BV (2008). The blood-brain barrier in health and chronic neurodegenerative disorders. Neuron.

[9] Tyas SL, Snowdon DA, Desrosiers MF, Riley KP, Markesbery WR (2007). Healthy ageing in the Nun study: definition and neuropathologic correlates. Age Ageing.

[10] van der Kolk AG, Zwanenburg JJ, Brundel M, Biessels GJ, Visser F, Luijten PR, Hendrikse J (2011). Intracranial vessel wall imaging at 7.0 T MRI. Stroke.

[11] Bouvy WH, Biessels GJ, Kuijf HJ, Kappelle LJ, Luijten PR, Zwanenburg JJ (2014). Visualization of perivascular spaces and perforating arteries with 7 T magnetic resonance imaging. Invest Radiol.

[12] Bouvy WH, Geurts LJ, Kuijf HJ, Luijten PR, Kappelle LJ, Biessels GJ, Zwanenburg JJ (2015). Assessment of blood flow velocity and pulsatility in cerebral perforating arteries with 7-T quantitative flow MRI. NMR Biomed.

[13] van Veluw SJ, Zwanenburg JJ, Engelen-Lee J, Spliet WG, Hendrikse J, Luijten PR (2013). In vivo detection of cerebral cortical microinfarcts with high-resolution 7T MRI. J Cereb Blood Flow Metab.

[14] van Veluw SJ, Hilal S, Kuijf HJ, Ikram MK, Xin X, Yeow TB, Venketasubramanian N, Biessels GJ, Chen C (2015). Cortical microinfarcts on 3T MRI: clinical correlates in memory-clinic patients. Alzheimers Dement..

[15] Biesbroek JM, Kuijf HJ, van der Graaf Y, Vincken KL, Postma A, Mali WP (2013). Association between subcortical vascular lesion location and cognition: a voxel-based and tract-based lesion-symptom mapping study. The SMART-MR study. PLoS ONE.

[16] Reijmer YD, Fotiadis P, Martinez-Ramirez S, Salat DH, Schultz A, Shoamanesh A, Ayres AM, Vashkevich A, Rosas D, Schwab K, Leemans A, Biessels GJ, Rosand J, Johnson KA, Viswanathan A, Gurol ME, Greenberg SM (2015). Structural network alterations and neurological dysfunction in cerebral amyloid angiopathy. Brain..

[17] Ances BM, Hammond DA (2014). Neuroimaging of HIV-associated neurocognitive disorders. Curr Opin HIV AIDS..

[18] Crystal HA, Weedon J, Holman S, Manly J, Valcour V, Cohen M, Anastos K, Liu C, Mack WJ, Golub E, Lazar J, Ho A, Kreek MJ, Kaplan RC (2011). Associations of cardiovascular variables and HAART with cognition in middle-aged HIV-infected and uninfected women. J Neurovirol..

[19] Baird AE (2010). Genetics and genomics of stroke: novel approaches. J Am Coll Cardiol.

[20] Baird AE, Soper SA, Pullagurla SR, Adamski MG (2015). Recent and near-future advances in nucleic acid-based diagnosis of stroke. Expert Rev Mol Diagn..

[21] Rosenberg GA, Bjerke M, Wallin A (2011). Multimodal markers of inflammation in the subcortical ischemic vascular disease type of vascular cognitive impairment. Stroke.

[22] Moore DF, Li H, Jeffries N, Wright V, Cooper RA, Elkahloun A, Gelderman MP, Zudaire E, Blevins G, Yu H, Goldin E, Baird AE (2005). Using peripheral blood mononuclear cells to determine a gene expression profile of acute ischemic stroke: a pilot investigation. Circulation.

[23] Tang Y, Xu H, Du X, Lit L, Walker W, Lu A, Ran R, Gregg JP, Reilly M, Pancioli A, Khoury JC, Sauerbeck LR, Carrozzella JA, Spilker J, Clark J, Wagner KR, Jauch EC, Chang DJ, Verro P, Broderick JP, Sharp FR (2006). Gene expression in blood changes rapidly in neutrophils and monocytes after ischemic stroke in humans: a microarray study. J Cereb Blood Flow Metab.

[24] Jickling GC, Xu H, Stamova B, Ander BP, Zhan X, Tian Y, Liu D, Turner RJ, Mesias M, Verro P, Khoury J, Jauch EC, Pancioli A, Broderick JP, Sharp FR (2010). Signatures of cardioembolic and large-vessel ischemic stroke. Ann Neurol..

[25] Adamski MG, Li Y, Wagner E, Yu H, Seales-Bailey C, Soper SA, Murphy M, Baird AE (2013). Next-generation qPCR for the high-throughput measurement of gene expression in multiple leukocyte subsets. J Biomol Screen.

[26] Pullagurla SR, Baird AE, Adamski MG, Soper SA (2015). Current and future bioanalytical approaches for stroke assessment. Bioanalysis..

[27] Wong CH, Jenne CN, Lee WY, Léger C, Kubes P (2011). Functional innervation of hepatic iNKT Cells is immunosuppressive following stroke. Science.

[28] Nadareishvili ZG, Li H, Wright V, Maric D, Warach S, Hallenbeck JM, Dambrosia J, Barker JL, Baird AE (2004). Elevated pro-Inflammatory CD4 + CD28. Lymphocytes and stroke recurrence and death. Neurology..

[29] Yan J, Read SJ, Henderson RD, Hull R, Sullivan JDO, McCombe PA, Greer JM (2012). Frequency and function of regulatory T cells after ischaemic stroke in humans. J Neuroimmunol.

[30] Mantani PT, Ljungcrantz I, Andersson L, Alm R, Hedblad B, Björkbacka H, Nilsson J, Fredrikson GN (2014). Circulating CD40 + and CD86 + B cell subsets demonstrate opposing associations with risk of stroke. Arterioscler Thromb Vasc Biol.

[31] Kleinschnitz C, Kraft P, Dreykluft A, Hagedorn I, Göbel K, Schuhmann MK, Langhauser F, Helluy X, Schwarz T, Bittner S, Mayer CT, Brede M, Varallyay C, Pham M, Bendszus M, Jakob P, Magnus T, Meuth SG, Iwakura Y, Zernecke A, Sparwasser T, Nieswandt B, Stoll G, Wiendl H (2012). Regulatory T cells are strong promoters of acute ischemic stroke in mice by inducing dysfunction of the cerebral microvasculature. Blood.

[32] Clarkson BD, Ling C, Shi Y, Harris MG, Rayasam A, Sun D, Salamat MS, Kuchroo V, Lambris JD, Sandor M, Fabry Z (2014). T Cell-Derived Interleukin (IL)-21 promotes brain injury following stroke in mice. J Exp Med.

[33] Adamski MG, Li Y, Wagner E, Yu H, Seales-Bailey C, Durkin H, Hao Q, Soper SA, Murphy M, Baird AE (2014). Pre-existing hypertension dominates γδT cell reduction in human ischemic stroke. PLoS ONE.

[34] Shichita T, Sugiyama Y, Ooboshi H, Sugimori H, Nakagawa R, Takada I, Iwaki T, Okada Y, Iida M, Cua DJ, Iwakura Y, Yoshimura A (2009). Pivotal role of cerebral interleukin-17-producing gammadelta T cells in the delayed phase of ischemic brain injury. Nat Med.

[35] Estruch R, Ros E, Salas-Salvado J, Covas MI, Corella D, Aros F, Gomez-Gracia E, Ruiz-Gutierrez V, Fiol M, Lapetra J, Lamuela-Raventos RM, Serra-Majem L, Pinto X, Basora J, Munoz MA, Sorli JV, Martinez JA, Martinez-Gonzalez MA (2013). Primary prevention of cardiovascular disease with a Mediterranean diet. N Engl J Med.

[36] Calton EK, James AP, Pannu PK, Soares MJ (2014). Certain dietary patterns are beneficial for the metabolic syndrome: reviewing the evidence. Nutr Res..

[37] Grosso G, Pajak A, Mistretta A, Marventano S, Raciti T, Buscemi S, Drago F, Scalfi L, Galvano F (2014). Protective role of the Mediterranean diet on several cardiovascular risk factors: evidence from Sicily, southern Italy. Nutr Metab Cardiovasc Dis..

[38] Scarmeas N, Stern Y, Mayeux R, Manly JJ, Schupf N, Luchsinger JA (2009). Mediterranean diet and mild cognitive impairment. Arch Neurol.

[39] de Waal H, Stam CJ, Lansbergen MM, Wieggers RL, Kamphuis PJ, Scheltens P, Maestu F, van Straaten EC (2014). The effect of souvenaid on functional brain network organisation in patients with mild Alzheimer’s disease: a randomised controlled study. PLoS ONE.

[40] Shah RC, Kamphuis PJ, Leurgans S, Swinkels SH, Sadowsky CH, Bongers A, Rappaport SA, Quinn JF, Wieggers RL, Scheltens P, Bennett DA (2013). The S-Connect study: results from a randomized, controlled trial of Souvenaid in mild-to-moderate Alzheimer’s disease. Alzheimers Res Ther.

[41] van Wijk N, Broersen LM, de Wilde MC, Hageman RJ, Groenendijk M, Sijben JW, Kamphuis PJ (2014). Targeting synaptic dysfunction in Alzheimer’s disease by administering a specific nutrient combination. J Alzheimers Dis.

[42] Braidy N, Muñoz P, Palacios AG, Castellano-Gonzalez G, Inestrosa NC, Chung RS, Sachdev P, Guillemin GJ (2012). Recent rodent models for Alzheimer’s disease: clinical implications and basic research. J Neural Transm..

[43] Haast RA, Gustafson DR, Kiliaan AJ (2012). Sex differences in stroke. J Cereb Blood Flow Metab.

[44] Andersen K, Launer LJ, Dewey ME, Letenneur L, Ott A, Copeland JR, Dartigues JF, Kragh-Sorensen P, Baldereschi M, Brayne C, Lobo A, Martinez-Lage JM, Stijnen T, Hofman A (1999). Gender differences in the incidence of AD and vascular dementia: The EURODEM studies. EURODEM Incidence Research Group. Neurology..

[45] Zekry D, Hauw JJ, Gold G (2002). Mixed dementia: epidemiology, diagnosis, and treatment. J Am Geriatr Soc.

[46] Masdeu JC, Kreisl WC, Berman KF (2012). The neurobiology of Alzheimer disease defined by neuroimaging. Curr Opin Neurol.

[47] Dijkhuizen RM, van der Marel K, Otte WM, Hoff EI, van der Zijden JP, van der Toorn A, van Meer MP (2012). Functional MRI and diffusion tensor imaging of brain reorganization after experimental stroke. Transl Stroke Res..

[48] Jansen D, Zerbi V, Janssen CI, Dederen PJ, Mutsaers MP, Hafkemeijer A, Janssen AL, Nobelen CL, Veltien A, Asten JJ, Heerschap A, Kiliaan AJ (2013). A longitudinal study of cognition, proton MR spectroscopy and synaptic and neuronal pathology in aging wild-type and AbetaPPswe-PS1dE9 mice. PLoS ONE.

[49] Zerbi V, Wiesmann M, Emmerzaal TL, Jansen D, van Beek M, Mutsaers MP, Beckmann CF, Heerschap A, Kiliaan AJ (2014). Resting-state functional connectivity changes in aging apoE4 and apoE-KO mice. J Neurosci.

[50] Rosenberg GA, Wallin A, Wardlaw JM, Markus HS, Montaner J, Wolfson L, Iadecola C, Zlokovic BV, Joutel A, Dichgans M, Duering M, Schmidt R, Korczyn AD, Grinberg LT, Chui HC, Hachinski V (2015). Consensus statement for diagnosis of subcortical small vessel disease. J Cereb Blood Flow Metab.

[51] Zhang R, Chopp M, Zhang ZG (2013). Oligodendrogenesis after cerebral ischemia. Front Cell Neurosci..

[52] Maki T, Liang AC, Miyamoto N, Lo EH, Arai K (2013). Mechanisms of oligodendrocyte regeneration from ventricular–subventricular zone-derived progenitor cells in white matter diseases. Front Cell Neurosci..

[53] Miyamoto N, Pham LD, Hayakawa K, Matsuzaki T, Seo JH, Magnain C, Ayata C, Kim KW, Boas D, Lo EH, Arai K (2013). Age-related decline in oligodendrogenesis retards white matter repair in mice. Stroke.

[54] Seo JH, Maki T, Maeda M, Miyamoto N, Liang AC, Hayakawa K, Pham LD, Suwa F, Taguchi A, Matsuyama T, Ihara M, Kim KW, Lo EH, Arai K (2014). Oligodendrocyte precursor cells support blood-brain barrier integrity via TGF-β signaling. PLoS ONE.

[55] Seo JH, Miyamoto N, Hayakawa K, Pham LD, Maki T, Ayata C, Kim KW, Lo EH, Arai K (2013). Oligodendrocyte precursors induce early blood-brain barrier opening after white matter injury. J Clin Invest..

[56] Barone FC, Rosenbaum DM, Zhou J, Crystal H (2009). Vascular cognitive impairment: dementia biology and translational animal models. Curr Opin Invest Drugs..

[57] Gorelick PB, Scuteri A, Black SE, Decarli C, Greenberg SM, Iadecola C, Launer LJ, Laurent S, Lopez OL, Nyenhuis D, Petersen RC, Schneider JA, Tzourio C, Arnett DK, Bennett DA, Chui HC, Higashida RT, Lindquist R, Nilsson PM, Roman GC, Sellke FW, Seshadri S, American Heart Association Stroke Council, Council on Epidemiology and Prevention, Council on Cardiovascular Nursing, Council on Cardiovascular Radiology and Intervention and Council on Cardiovascular Surgery and Anesthesia (2011). Vascular contributions to cognitive impairment and dementia: a statement for healthcare professionals from the american heart association/american stroke association. Stroke.

[58] Hachinski V, Iadecola C, Petersen RC, Breteler MM, Nyenhuis DL, Black SE, Powers WJ, DeCarli C, Merino JG, Kalaria RN, Vinters HV, Holtzman DM, Rosenberg GA, Wallin A, Dichgans M, Marler JR, Leblanc GG (2006). National Institute of Neurological Disorders and Stroke-Canadian stroke Network Vascular Cognitive Impairment Harmonization Standards. Stroke.

[59] Jiwa NS, Garrard P, Hainsworth AH (2010). Experimental models of vascular dementia and vascular cognitive impairment: a systematic review. J Neurochem.

[60] Rosenberg GA (2009). Inflammation and white matter damage in vascular cognitive impairment. Stroke.

[61] Jalal FY, Yang Y, Thompson J, Lopez AC, Rosenberg GA (2012). Myelin loss associated with neuroinflammation in hypertensive rats. Stroke.

[62] Choi JY, Cui Y, Kim BG (2015). Interaction between hypertension and cerebral hypoperfusion in the development of cognitive dysfunction and white matter pathology in rats. Neuroscience.

[63] Gelber RP, Launer LJ, White LR (2012). The Honolulu-Asia aging study: epidemiologic and neuropathologic research on cognitive impairment. Curr Alzheimer Res.

[64] Harris TB, Launer LJ, Eiriksdottir G, Kjartansson O, Jonsson PV, Sigurdsson G, Thorgeirsson G, Aspelund T, Garcia ME, Cotch MF, Hoffman HJ, Gudnason V (2007). Age, gene/environment susceptibility-reykjavik study: multidisciplinary applied phenomics. Am J Epidemiol.

[65] Torre JCDL (2004). Is Alzheimer’s disease a neurodegenerative or a vascular disorder? Data, dogma, and dialectics. Lancet Neurol.

[66] Kalaria RN (2012). CVD and mechanisms of cognitive impairment: evidence from clinicopathological studies in humans. Stroke.

[67] Kling MA, Trojanowski JQ, Wolk DA, Lee VM, Arnold SE (2013). Vascular disease and dementias: paradigm shifts to drive research in new directions. Alzheimers Dement..

[68] Misiak B, Leszek J, Kiejna A (2012). Metabolic syndrome, mild cognitive impairment and Alzheimer’s disease–the emerging role of systemic low-grade inflammation and adiposity. Brain Res Bull.

[69] Chertkow H, Feldman HH, Jacova C, Massoud F (2013). Definitions of dementia and predementia states in Alzheimer’s disease and vascular cognitive impairment: consensus from the Canadian conference on diagnosis of dementia. Alzheimers Res Ther.

[70] Luchsinger JA (2012). Type 2 diabetes and cognitive impairment: linking mechanisms. J Alzheimers Dis.

[71] Sorbi S, Hort J, Erkinjuntti T, Fladby T, Gainotti G, Gurvit H, Nacmias B, Pasquier F, Popescu BO, Rektorova I, Religa D, Rusina R, Rossor M, Schmidt R, Stefanova E, Warren JD, Scheltens P (2012). EFNS Scientist Panel on Dementia and Cognitive Neurology. EFNS-ENS guidelines on the diagnosis and management of disorders associated with dementia. Eur J Neurol.

[72] Andrieu S, Aboderin I, Baeyens JP, Beard J, Benetos A, Berrut G, Brainin M, Cha HB, Chen LK, Du P, Forette B, Forette F, Franco A, Fratiglioni L, Gillette-Guyonnet S, Gold G, Gomez F, Guimaraes R, Gustafson D, Khachaturian A, Luchsinger J, Mangialasche F, Mathiex-Fortunet H, Michel JP, Richard E, Schneider LS, Solomon A, Vellas B (2011). IAGG workshop: health promotion program on prevention of late onset dementia. J Nutr Health Aging..

[73] Sachdev P, Kalaria R, Brien JO, Skoog I, Alladi S, Black SE, Blacker D, Blazer DG, Chen C, Chui H, Ganguli M, Jellinger K, Jeste DV, Pasquier F, Paulsen J, Prins N, Rockwood K, Roman G, Scheltens P (2014). Internationlal Society for Vascular Behavioral and Cognitive Disorders. Diagnostic criteria for vascular cognitive disorders: a VASCOG statement. Alzheimer Dis Assoc Disord.

[74] Wallin A, Nordlund A, Jonsson M, Blennow K, Zetterberg H, Öhrfelt A, Stålhammar J, Eckerström M, Carlsson M, Olsson E, Göthlin M, Svensson J, Rolstad S, Eckerström C, Bjerke M. Alzheimer’s disease-subcortical vascular disease spectrum in a hospital-based setting: overview of results from the Gothenburg MCI and dementia studies. J Cereb Blood Flow Metab. 2015. [Epub ahead of print]. 10.1038/jcbfm.2015.148 PMC4702291 26219595

